# Rivaroxaban treatment for asymptomatic venous thromboembolism: insights from the J’xactly study

**DOI:** 10.1186/s12959-023-00528-w

**Published:** 2023-08-21

**Authors:** Shohei Migita, Yasuo Okumura, Ikuo Fukuda, Mashio Nakamura, Norikazu Yamada, Morimasa Takayama, Hideaki Maeda, Takeshi Yamashita, Takanori Ikeda, Makoto Mo, Tsutomu Yamazaki, Atsushi Hirayama, on behalf of the J’xactly Investigators

**Affiliations:** 1 Division of Cardiology, Department of Medicine, Nihon University School of Medicine, Ohyaguchi-kamicho, Itabashi-ku, Tokyo, 173-8610 Japan; 2 Department of Cardiology, Keimeikai Yokawa Hospital, Miki, Japan; 3 Nakamura Medical Clinic, Kuwana, Japan; 4 Department of Cardiology, Kuwana City Medical Center, Kuwana, Japan; 5 Department of Cardiology, Sakakibara Heart Institute, Fuchu, Japan; 6 Department of Heart and Vascular Center, Ukima Central Hospital, Tokyo, Japan; 7 Department of Cardiovascular Medicine, The Cardiovascular Institute, Tokyo, Japan; 8 Department of Cardiovascular Medicine, Toho University Faculty of Medicine, Tokyo, Japan; 9 Department of Cardiovascular Surgery, Yokohama Minami Kyosai Hospital, Yokohama, Japan; 10 Innovation and Research Support Center, International University of Health and Welfare, Tokyo, Japan; 11 Department of Cardiology, Osaka Police Hospital, Osaka, Japan

**Keywords:** Anticoagulant, Bleeding, Recurrence, Rivaroxaban, Venous thromboembolism

## Abstract

**Background:**

An established treatment strategy for asymptomatic pulmonary embolism (PE) or deep vein thrombosis (DVT) remains uncertain in Japan; therefore, in this study, we clarify the characteristics and outcomes of symptomatic compared to asymptomatic patients with PE or DVT.

**Methods:**

This prospective, multicenter sub-analysis of the J’xactly study in Japan included 1,016 patients (mean age, 68; 41% male) with venous thromboembolism (VTE) treated with rivaroxaban.

**Results:**

Asymptomatic PE patients (47% of PE patients) were more likely to have active cancer and asymptomatic proximal DVT at lower severity than symptomatic PE patients, despite no differences in age, sex, or the proportion receiving intensive 30 mg/day-rivaroxaban. Patients with asymptomatic DVT (34% of DVT patients) were older, had higher rates of female sex, active cancer, and distal DVT, and received shorter, less intense rivaroxaban treatment. Incidences did not differ between asymptomatic and symptomatic PE patients for recurrent symptomatic VTE (hazard ratio [HR], 0.60; 95% confidence interval [CI], 0.22–1.62; *P* = 0.31) or major bleeding (HR, 0.68; 95% CI, 0.20–2.33; *P* = 0.58), nor between asymptomatic and symptomatic DVT patients for recurrent symptomatic VTE (HR, 0.56; 95% CI, 0.23–1.40; *P* = 0.21) and major bleeding (HR, 1.47; 95% CI, 0.54–3.97; *P* = 0.45).

**Conclusions:**

The real-world composite adverse event rate for treatment with rivaroxaban, as physician-adjusted for dose and duration, was similar for asymptomatic and symptomatic patients regardless of the presence of PE or DVT, suggesting a favorable safety profile for potential rivaroxaban treatment for asymptomatic VTE.

## Background

Venous thromboembolism (VTE), in the form of pulmonary embolism (PE) or deep vein thrombosis (DVT), is a common acute cardiovascular disease [1, 2] and a major medical problem worldwide [3]. PE can be detected incidentally on routine chest and abdominal computed tomography (CT) imaging studies [4, 5]. The detection of asymptomatic PE has increased with the introduction of multidirectional CT scanners that can better delineate the pulmonary arteries down to the segmental level [6]. The largest meta-analysis to date investigated more than 10,000 patients with VTE through 2009 and reported the incidence of asymptomatic PE to be 2.6% (95% confidence interval [CI] 1.9–3.4%). This was even higher in patients with VTE risk factors such as malignancy and hospitalization [7]. The optimal treatment strategy for incidentally detected asymptomatic PE remains controversial [8], although some data supports the treatment of incident PE in patients with known VTE risk factors such as malignancy [9–11]. The detection of asymptomatic distal DVT has also increased in recent years. It is considered to convey a lower risk than symptomatic distal DVT [12, 13]. Little value has been associated with uniformly administering therapeutic doses of anticoagulation for screen-detected distal DVT. Anticoagulation therapy is used for proximal DVT and PE; however, the safety and advantage of distal DVT anticoagulation treatment remain to be established.

Therefore, this study examines the background and clinical outcomes in 1,039 patients with acute symptomatic and asymptomatic DVT only (DVT group) or PE with or without DVT (PE group) treated with rivaroxaban. We used the J’xactly study (a Japanese registry) data on the efficacy and safety of rivaroXAban for the prevention of reCurrence in deep vein Thrombosis and puLmonarY Embolism patients (University Hospital Medical Information Network Clinical Trials Registry, UMIN000025072).

## Methods

### Study population

**Fig. 1:**
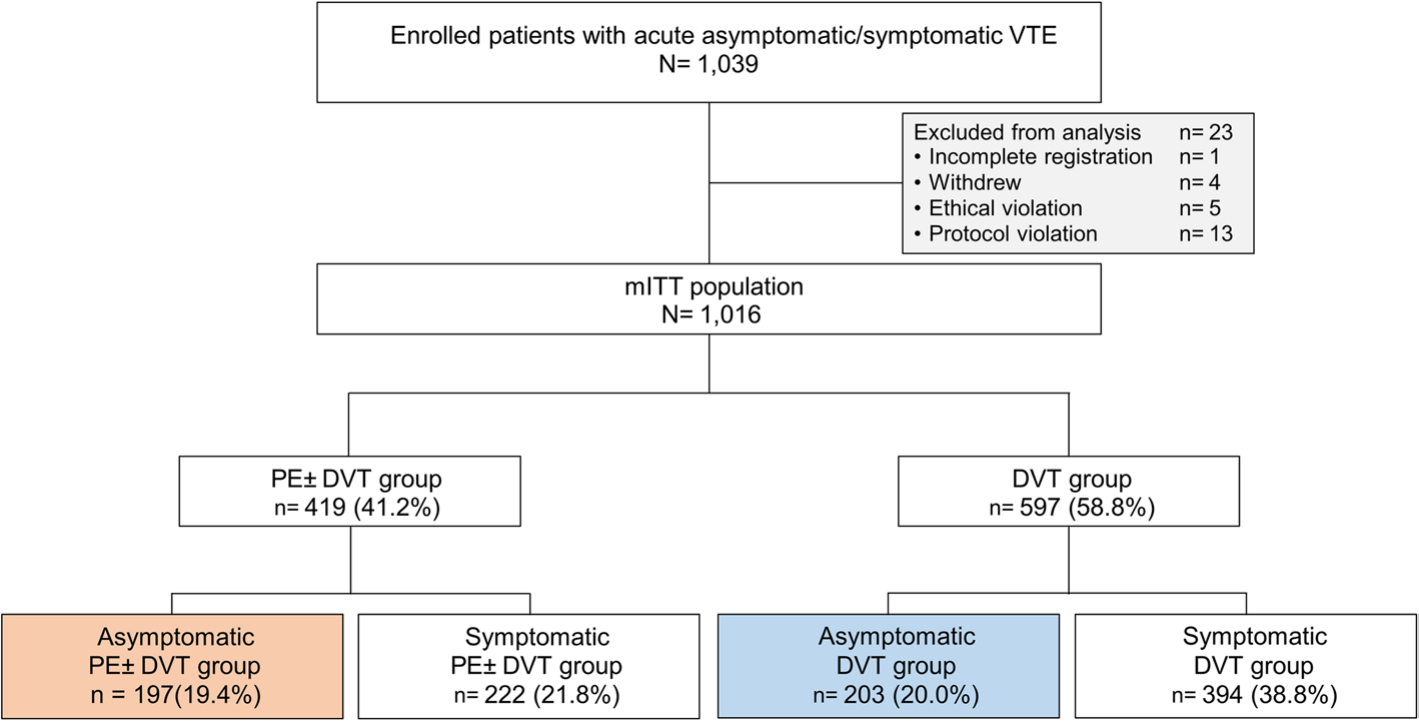
Flowchart of patient selection and stratification by symptoms. DVT, deep venous thrombosis; mITT, modified intention-to-treat; PE, pulmonary embolism; VTE, venous thromboembolism

The J’xactly study was a multicenter, prospective, observational cohort study in which patients diagnosed with acute symptomatic and asymptomatic DVT, PE, or both and prescribed rivaroxaban for the treatment and prevention of VTE were enrolled from December 2016 to April 2018. Details of the study design, data collection process, and baseline characteristics of the study population have been previously described [14, 15]. The key exclusion criteria were contraindications to rivaroxaban; chronic thromboembolic pulmonary hypertension (CTEPH), except for CTEPH plus acute PE or DVT; active bleeding. All the patients provided written informed consent to participate in this study. All eligible patients were enrolled in the study within 3 weeks of starting rivaroxaban for the treatment and prevention of VTE. Data were collected until the end of the follow-up period (November 2019), regardless of whether rivaroxaban was continued, discontinued, or terminated according to either the patient’s preference or the physician’s discretion. In this study, a total of 1,016 patients evaluable by modified intention-to-treat (mITT) were included. First, patients were stratified according to the presence of PE with or without DVT (PE group) and DVT only (DVT group). Both the PE and DVT groups were evaluated for the efficacy and safety endpoints, which were compared between the symptomatic and asymptomatic patients (Fig. 1). Symptomatic patients in the PE group were defined by the presence of PE-related symptoms, including dyspnea, tachypnea, chest pain, cold sweats, fainting, palpitation, tachycardia, cough, wheezing, bloody sputum, shock, hypotension, and fever. Symptomatic patients in the DVT group were defined by the presence of DVT-related symptoms, including pain, swelling, superficial venous aggravation, color changes due to congestion, tautness, edema, tenderness, and Homan’s sign of the lower extremities. PE severity was stratified according to Japanese guidelines [2] as either cardiac arrest or collapse, massive, sub-massive or non-massive PE, in addition to the criteria described above. DVT was classified by the thrombus location as either proximal (thrombus located proximal to or involving the popliteal vein) or distal (thrombus located distal to the popliteal vein). Data regarding the initial dose of rivaroxaban (standard dosage: 30 mg/day; under-dosages: 20, 15, or 10 mg/day) were also recorded.

The J’xactly study was conducted in accordance with the principles of the Declaration of Helsinki and all applicable legal and regulatory requirements in Japan. The study protocols and related documentation were reviewed and approved by the Institutional Review Board (IRB) of Nihon University Itabashi Hospital. All the participating institutions provided ethical approval.

### Endpoints

The primary effectiveness outcome was the recurrence or aggravation of symptomatic VTE during the follow-up period [14, 15]. VTE was defined according to the established diagnostic criteria [16]. The primary safety outcome was the occurrence of a major bleeding during the treatment period and up to 2 days after rivaroxaban discontinuation. Major bleeding was defined according to the International Society on Thrombosis and Hemostasis criteria [17]. Secondary outcomes included the recurrence or aggravation of symptomatic DVT and PE, death from any cause, death related to VTE and cardiovascular disease (CVD), vascular events (acute coronary syndrome [ACS] or ischemic stroke), and non-major bleeding. Clinically relevant events were also evaluated as composite outcomes, in which each component (recurrent VTE, ACS, ischemic stroke, death from any cause, and major bleeding events) was weighted equally. An independent, blinded clinical events committee adjudicated the outcomes.

### Statistical analysis

The mITT population was used for the effectiveness calculations, which included all enrolled patients except those who were excluded from the study. The on-treatment population included all patients treated with at least one dose of rivaroxaban. It was used for safety assessments. Continuous variables are reported as mean ± standard deviation, and categorical variables are reported as the number and percentage of patients. The symptomatic and asymptomatic PE and DVT groups were compared using the *t*-test for continuous variables and the chi-square test for categorical variables. The Kaplan–Meier method was used to estimate the cumulative event rates, with the incidence rates in each treatment group demonstrated as percentages per patient-year. A Cox proportional hazards regression model was used to compare outcomes between the two groups, and the results were expressed as hazard ratios (HRs) with 95% CIs. All statistical analyses were performed using JMP Pro 11 software (SAS Institute, Cary, NC, USA). Statistical significance was set at *P* < 0.05.

## Results

### Comparison of baseline patient characteristics in symptomatic and asymptomatic PE and DVT groups

Among the 419 patients in the PE group, 197 (47.0%) were asymptomatic and 222 (53.0%) were symptomatic, while of the 597 patients with DVT only, 203 (34.0%) were asymptomatic, and 394 (66.0%) were symptomatic. The baseline characteristics of the asymptomatic and symptomatic patients in the PE and DVT groups are summarized in Table 1. No differences in age, female sex, body weight, or creatinine clearance (CrCL) were observed between the asymptomatic and symptomatic PE groups. However, the asymptomatic PE group demonstrated a lower severity of illness than the symptomatic PE group overall, which was reflected by a lower heart rate (81.0 ± 14.8 vs. 93.9 ± 20.9 beats per minute; *P* < 0.001), higher oxygen saturation (97 ± 2 vs. 94 ± 6%; *P* < 0.001), higher rate of non-massive PE (82.2 vs. 38.7%; *P* < 0.001), smaller mean right ventricular (RV) diameter (32.6 ± 6.3 vs. 38.5 ± 10.4 mm; *P* < 0.001), lower RV/left ventricular diameter ratio (0.81 ± 0.21 vs. 1.06 ± 0.41; *P* < 0.001), and fewer comorbidities. Outpatient status, active cancer, history of recent surgery, use of nonsteroidal anti-inflammatory drugs, and proximal and asymptomatic DVT were more prevalent in the asymptomatic than the symptomatic PE group. Prior anticoagulation therapy was less prevalent in the asymptomatic than the symptomatic PE group. Although no differences were found in the initial dose of rivaroxaban dose, the total duration of rivaroxaban treatment tended to be longer in the asymptomatic compared to the symptomatic PE group (442 [183–661] vs. 361 [165–630] days; *P* = 0.08).

**Table 1:** Baseline characteristics of patients stratified by baseline symptoms

	PE with/without DVT				DVT			
Variable	Total	Asymptomatic	Symptomatic	*P*-Value	Total	Asymptomatic	Symptomatic	*P*-Value
	*n* = 419	*n* = 197	*n* = 222		*n* = 597	*n* = 203	*n* = 394	
Age (years)	66 ± 15	65 ± 14	66 ± 15	0.32	70 ± 15	72 ± 12	69 ± 16	0.017
≥ 75 years	137 (32.7%)	58 (29.4%)	79 (35.6%)	0.21	253 (42.4%)	88 (43.3%)	165 (41.9%)	0.79
Female sex	220 (52.5%)	94 (47.7%)	126 (56.8%)	0.08	379 (63.5%)	151 (74.4%)	228 (57.9%)	< 0.001
Body weight (kg)	63.0 ± 14.9	62.7 ± 13.8	63.3 ± 15.9	0.69	58.3 ± 12.9	55.5 ± 11.7	59.9 ± 13.5	< 0.001
< 50 kg	80 (19.1%)	33 (16.8%)	47 (21.2%)	0.26	142 (23.8%)	63 (31.0%)	79 (20.1%)	0.003
Body mass index (kg/m^2^)	24.2 ± 4.4	23.8 ± 3.9	24.5 ± 4.8	0.09	23.5 ± 4.0	23.1 ± 3.9	23.8 ± 4.1	0.05
Heart rate (beats per minute)	87.9 ± 19.4	81.0 ± 14.8	93.9 ± 20.9	< 0.001	78.3 ± 13.5	77.3 ± 13.6	79.0 ± 13.5	0.21
Systolic blood pressure (mmHg)	129.9 ± 23.5	129.8 ± 19.8	130.0 ± 26.3	0.93	128.8 ± 18.1	126.5 ± 15.7	130.1 ± 19.3	0.036
SpO_2_ (%)	95 ± 5	97 ± 2	94 ± 6	< 0.001	97 ± 2	97 ± 2	97 ± 2	0.26
Outpatient	112 (26.7%)	62 (31.5%)	50 (22.5%)	0.046	310 (51.9%)	57 (28.1%)	253 (64.2%)	< 0.001
CrCL (mL/minute)	82.1 ± 37.6	84.5 ± 33.7	79.9 ± 40.6	0.21	77.2 ± 35.0	74.0 ± 29.2	79.0 ± 37.8	0.045
<50 mL/minute	71 (16.9%)	29 (14.7%)	42 (18.9%)	0.30	199 (33.3%)	69 (34.0%)	130 (33.0%)	0.74
D-dimer (µg/mL)	10.0 (5.5–19.0)	9.3 (5.3–17.5)	10.7 (5.7–19.9)	0.13	7.6 (3.5–15.1)	7.9 (3.5–14.2)	7.6 (3.4–16.2)	0.84
Medical history								
Hypertension	171 (40.8%)	71 (36.0%)	98 (44.1%)	0.11	211 (35.3%)	69 (34.0%)	142 (36.0%)	0.65
Diabetes mellitus	54 (12.9%)	27 (13.7%)	27 (12.2%)	0.66	64 (10.7%)	28 (13.8%)	36 (9.1%)	0.09
Heart failure	18 (4.3%)	3 (1.5%)	15 (6.8%)	0.008	16 (2.7%)	7 (3.4%)	9 (2.3%)	0.43
Atrial fibrillation	16 (3.8%)	5 (2.5%)	11 (5.0%)	0.21	10 (1.7%)	6 (3.0%)	4 (1.0%)	0.10
Coronary artery disease	19 (4.5%)	5 (2.5%)	14 (6.3%)	0.10	26 (4.4%)	9 (4.4%)	17 (4.3%)	1.00
Chronic heart and lung disease	27 (6.4%)	7 (3.6%)	20 (9.0%)	0.028	20 (3.4%)	9 (4.4%)	11 (2.8%)	0.34
Previous stroke	28 (6.7%)	13 (6.6%)	15 (6.8%)	1.00	45 (7.5%)	16 (7.9%)	29 (7.4%)	0.87
Previous atrial fibrillation	8 (1.9%)	2 (1.0%)	6 (2.7%)	0.29	15 (2.5%)	7 (3.4%)	8 (2.0%)	0.41
Risk factor								
Active cancer	88 (21.0%)	54 (27.4%)	34 (15.3%)	0.003	105 (17.6%)	55 (27.1%)	50 (12.7%)	< 0.001
Recent surgery	78 (18.6%)	48 (24.4%)	30 (13.5%)	0.006	182 (30.5%)	113 (55.7%)	69 (17.5%)	< 0.001
Recent injury	31 (7.4%)	14 (7.1%)	17 (7.7%)	0.85	62 (10.4%)	33 (16.3%)	29 (7.4%)	0.001
Inactivity	130 (31.0%)	64 (32.5%)	66 (29.7%)	0.60	237 (39.7%)	119 (58.6%)	118 (29.9%)	< 0.001
Thrombophilia	21 (5.0%)	10 (5.1%)	11 (5.0%)	1.00	17 (2.8%)	4 (2.0%)	13 (3.3%)	0.44
Previous VTE	28 (6.7%)	13 (6.6%)	15 (6.8%)	1.00	55 (9.2%)	16 (7.9%)	39 (9.9%)	0.46
Concomitant medications								
Antiplatelet agents	38 (9.1%)	18 (9.1%)	20 (9.0%)	1.00	66 (11.1%)	28 (13.8%)	38 (9.6%)	0.13
Estrogen preparations	7 (1.7%)	3 (1.5%)	4 (1.8%)	1.00	16 (2.7%)	1 (0.5%)	15 (3.8%)	0.016
Anticancer agents	43 (10.3%)	20 (10.2%)	23 (10.4%)	1.00	45 (7.5%)	21 (10.3%)	24 (6.1%)	0.07
NSAIDs	61 (14.6%)	38 (19.3%)	23 (10.4%)	0.012	135 (22.6%)	72 (35.5%)	63 (16.0%)	< 0.001
DVT	320 (76.4%)	164 (83.2%)	156 (70.3%)	0.002				
Proximal	235 (56.1%)	133 (67.5%)	102 (45.9%)	< 0.001	294 (49.3%)	53 (26.1%)	241 (61.2%)	< 0.001
Distal	85 (20.3%)	31 (15.7%)	54 (24.3%)	0.038	303 (50.8%)	150 (73.9%)	153 (38.8%)	< 0.001
Localization of right DVT	196 (46.8%)	97 (49.2%)	99 (44.6%)	0.38	332 (55.6%)	137 (67.5%)	195(49.5%)	< 0.001
Localization of left DVT	194 (46.3%)	97 (49.2%)	97 (43.7%)	0.28	402 (67.3%)	124 (61.1%)	278 (70.6%)	0.021
Localization of both DVT	70 (16.7%)	30 (15.2%)	40 (18.0%)	0.51	137 (22.9%)	58 (28.6%)	79 (20.1%)	0.024
Asymptomatic DVT	205 (48.9%)	125 (63.5%)	80 (36.0%)	< 0.001				
PE								
Cardiac arrest or Collapse or Massive	23 (5.5%)	0 (0.0%)	23 (10.4%)	< 0.001				
Sub-massive or Non-massive	371 (88.5%)	175 (88.8%)	196 (88.3%)	0.88				
Non-massive	248 (59.2%)	162 (82.2%)	86 (38.7%)	< 0.001				
Severer than Sub-massive	146 (34.8%)	13 (6.6%)	133 (59.9%)	< 0.001				
RV pressure indexes								
Mean RV diameter (mm)	36.1 ± 9.4	32.6 ± 6.3	38.5 ± 10.4	< 0.001				
RV/LV diameter ratio	0.96 ± 0.36	0.81 ± 0.21	1.06 ± 0.41	< 0.001				
Prior treatment								
Anticoagulation therapy	171 (40.8%)	59 (29.9%)	112 (50.5%)	< 0.001	93 (15.6%)	24 (11.8%)	69 (17.5%)	0.07
Inferior vena cava filter	40 (9.5%)	23 (11.7%)	17 (7.7%)	0.18	47 (7.9%)	6 (3.0%)	41 (10.4%)	0.001
Thrombolytic therapy	33 (7.9%)	11 (5.6%)	22 (9.9%)	0.10	14 (2.3%)	0 (0.0%)	14 (3.6%)	0.004
Catheterization	4 (1.0%)	2 (1.0%)	2 (0.9%)	1.00	8 (1.3%)	0 (0.0%)	8 (2.0%)	0.06
Pulmonary thrombus removal	1 (0.2%)	0 (0.0%)	1 (0.5%)	1.00	0 (0.0%)	0 (0.0%)	0 (0.0%)	-
PCPS	3 (0.7%)	0 (0.0%)	3 (1.4%)	0.25	0 (0.0%)	0 (0.0%)	0 (0.0%)	-
Others	14 (3.3%)	5 (2.5%)	9 (4.1%)	0.43	12 (2.0%)	2 (1.0%)	10 (2.5%)	0.36
Initial rivaroxaban treatment								
DOSE (mg/day)				0.37				0.004
30 mg/day	341 (81.4%)	154 (78.2%)	187 (84.2%)		326 (54.6%)	90 (44.3%)	236 (59.9%)	
20 mg/day	6 (1.4%)	4 (2.0%)	2 (0.9%)		16 (2.7%)	7 (3.4%)	9 (2.3%)	
15 mg/day	64 (15.3%)	34 (17.3%)	30 (13.5%)		218 (36.5%)	92 (45.3%)	126 (32.0%)	
10 mg/day	8 (1.9%)	5 (2.5%)	3 (1.4%)		37 (6.2%)	14 (6.9%)	23 (5.8%)	
Treatment duration, days								
Median (IQR)	388 (176–643)	442 (183–661)	361 (165–630)	0.08	282 (106–619)	106 (68–259)	338 (111–637)	< 0.001

Data are shown as n (%), median (interquartile range), or mean ± standard deviation, unless otherwise stated

*CrCL *Creatinine clearance, *DVT *Deep vein thrombosis, *IQR *Interquartile range, *LV *Left ventricle, *NSAIDs *Nonsteroidal anti-inflammatory drugs, *PCPS *Percutaneous cardiopulmonary support, *PE *Pulmonary embolism, *RV *Right ventricle, *SD *Standard deviation, *SpO*_2 _Oxygen saturation, *VTE *Venous thromboembolism

Asymptomatic patients were older (72 ± 12 vs. 69 ± 16 years; *P* = 0.017) and more often female (74.4 vs. 57.9%; *P* < 0.001) than symptomatic patients in the DVT group. Asymptomatic patients also had lower body weight (55.5 ± 11.7 vs. 59.9 ± 13.5 kg; *P* < 0.001) and lower CrCL (74.0 ± 29.2 vs. 79.0 ± 37.8 mL/min; *P* = 0.045) as compared with symptomatic DVT group. The asymptomatic DVT group more often had active cancer complications, recent surgery, and recent trauma as a cause of VTE than the symptomatic DVT group. Moreover, this group was more likely to use nonsteroidal anti-inflammatory drugs and was more frequently diagnosed during hospitalization (71.9 vs. 35.8%; *P* < 0.001) than the symptomatic DVT group. The asymptomatic DVT group had a higher rate of distal DVT (73.9% vs. 38.8%; *P* < 0.001) than the symptomatic DVT group. The asymptomatic DVT group more often initially received a reduced dose (20, 15, or 10 mg) of rivaroxaban (55.7 vs. 40.1%; *P* < 0.001) with a shorter treatment duration (106 [68–259] vs. 338 [111–637] days; *P* < 0.001).

### Clinical outcomes in asymptomatic and symptomatic PE and DVT groups

**Fig. 2:**
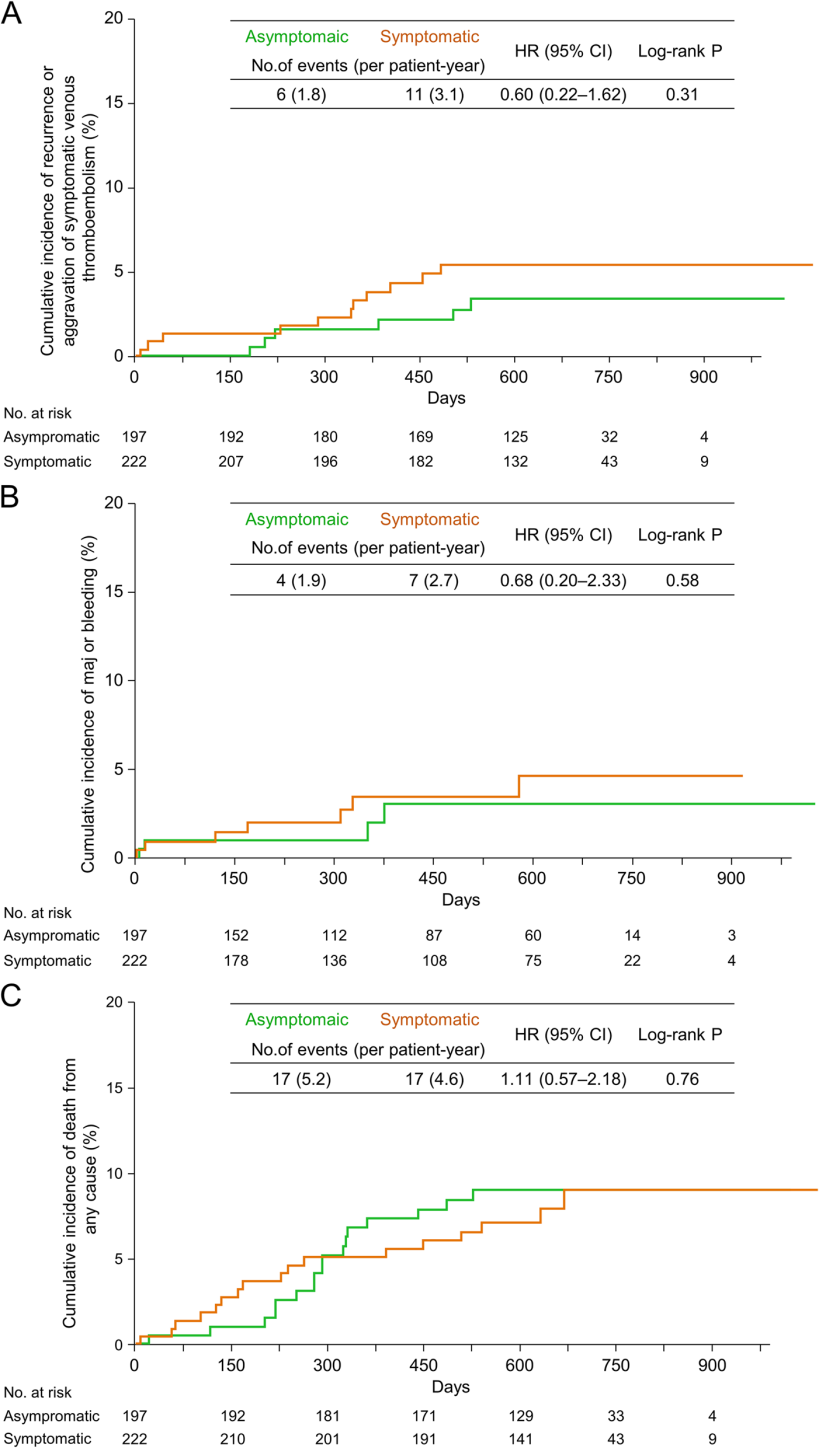
Results of clinical events in the PE group. Kaplan–Meier curves showing the cumulative incidence of (**A**) recurrence or aggravation of symptomatic VTE, (**B**) major bleeding, and (**C**) death from any cause in the PE group. CI, confidence interval; HR, hazard ratio; PE, pulmonary embolism; VTE, venous thromboembolism

During the median follow-up period of 21.3 months (interquartile range, 18.1–24.2 months), a recurrence or aggravation of symptomatic VTE was reported in 6 (3.0%) in the asymptomatic PE group versus 11 (5.0%) in the symptomatic PE group (1.8 vs. 3.1 events per patient-year; HR, 0.60; 95% CI, 0.22–1.62; *P* = 0.31, Fig. 2A). No difference in major bleeding events was noted between asymptomatic and symptomatic PE groups (1.9 vs. 2.7 events per patient-year; HR, 0.68; 95% CI, 0.20–2.33; *P* = 0.58, Fig. 2B). However, the incidence of minor bleeding was significantly lower in the asymptomatic than the symptomatic PE group (3.4 vs. 9.5 events per patient-year; HR, 0.36; 95% CI, 0.15–0.83; *P* = 0.004) (Table 2). No incidents of fatal bleeding occurred in either group (Table 2). The occurrence of death from any cause (5.2 vs. 4.6 events per patient-year; HR, 1.11; 95% CI, 0.57–2.18; *P* = 0.76, Fig. 2C) and clinically relevant events was similar (7.8 vs. 9.5 events per patient-year; HR, 0.83; 95% CI, 0.49–1.39; *P* = 0.48) in the two groups (Table 2).

**Table 2:** Clinical outcomes of patients stratified by baseline symptoms

	PE with/without DVT				DVT			
	Asymptomatic	Symptomatic	HR (95% CI)	*P*-Value	Asymptomatic	Symptomatic	HR (95% CI)	*P*-Value
	*n* = 197	*n* = 222			*n* = 203	*n* = 394		
Recurrence or aggravation of symptomatic VTE	1.8 (0.7–4.0)	3.1 (1.5–5.5)	0.60 (0.22–1.62)	0.31	1.7 (0.6–3.8)	3.1 (1.9–4.8)	0.56 (0.23–1.40)	0.21
Recurrence or aggravation of symptomatic PE	0.9 (0.2–2.7)	2.5 (1.1–4.5)	0.37 (0.10–1.35)	0.12	0.3 (0.0–1.6)	1.1 (0.4–2.2)	0.26 (0.03–2.15)	0.18
Recurrence or aggravation of symptomatic DVT	1.2 (0.3–3.2)	0.8 (0.2–2.4)	1.49 (0.33–6.67)	0.60	1.4 (0.5–3.4)	2.2 (1.2–3.6)	0.68 (0.24–1.88)	0.45
Acute coronary syndrome	0	0.3 (0.0–1.5)	-	0.35	0.3 (0.0–1.6)	0.2 (0.0–0.8)	1.90 (0.12–30.36)	0.64
Ischemic stroke	1.2 (0.3–3.1)	1.1 (0.3–2.8)	1.11 (0.28–4.45)	0.88	0	0	-	-
Death from any cause	5.2 (3.0–8.3)	4.6 (2.7–7.4)	1.11 (0.57–2.18)	0.76	6.5 (4.1–9.8)	5.6 (4.0–7.7)	1.17 (0.69–1.96)	0.56
Death related to VTE	0.6 (0.1–2.2)	0.5 (0.1–2.0)	1.11 (0.16–7.89)	0.92	0.3 (0.0–1.6)	0.5 (0.1–1.4)	0.62 (0.06–5.94)	0.67
Death related to CVD	0.9 (0.2–2.7)	0.5 (0.1–2.0)	1.67 (0.28–9.99)	0.57	0.3 (0.0–1.6)	1.1 (0.4–2.2)	0.27 (0.03–2.19)	0.19
Major bleeding	1.9 (0.5–4.9)	2.7 (1.1–5.7)	0.68 (0.20–2.33)	0.58	5.2 (1.9–11.4)	3.0 (1.5–5.2)	1.47 (0.54–3.97)	0.45
Minor bleeding	3.4 (1.4–7.0)	9.5 (6.0–14.3)	0.36 (0.15–0.83)	0.004	12.4 (6.8–20.8)	9.1 (6.3–12.6)	1.03 (0.55–1.92)	0.94
Fatal bleeding	0	0	-	-	0	0.5 (0.1–1.3)	-	0.21
Clinically relevant events	7.8 (5.1–11.6)	9.5 (6.5–13.3)	0.83 (0.49–1.39)	0.48	9.9 (6.8–13.9)	9.4 (7.2–12.1)	1.05 (0.69–1.61)	0.82

Data are shown as % per patient-year, unless otherwise stated

Clinically relevant events were evaluated as a composite outcome, in which each component (recurrent VTE, acute coronary syndrome, ischemic stroke, death from any cause, and major bleeding events) was weighted equally. *CI *Confidence interval, *CVD *Cardiovascular disease, *DVT *Deep vein thrombosis, *HR *Hazard ratio, *PE *Pulmonary embolism, *VTE *Venous thromboembolism

**Fig. 3:**
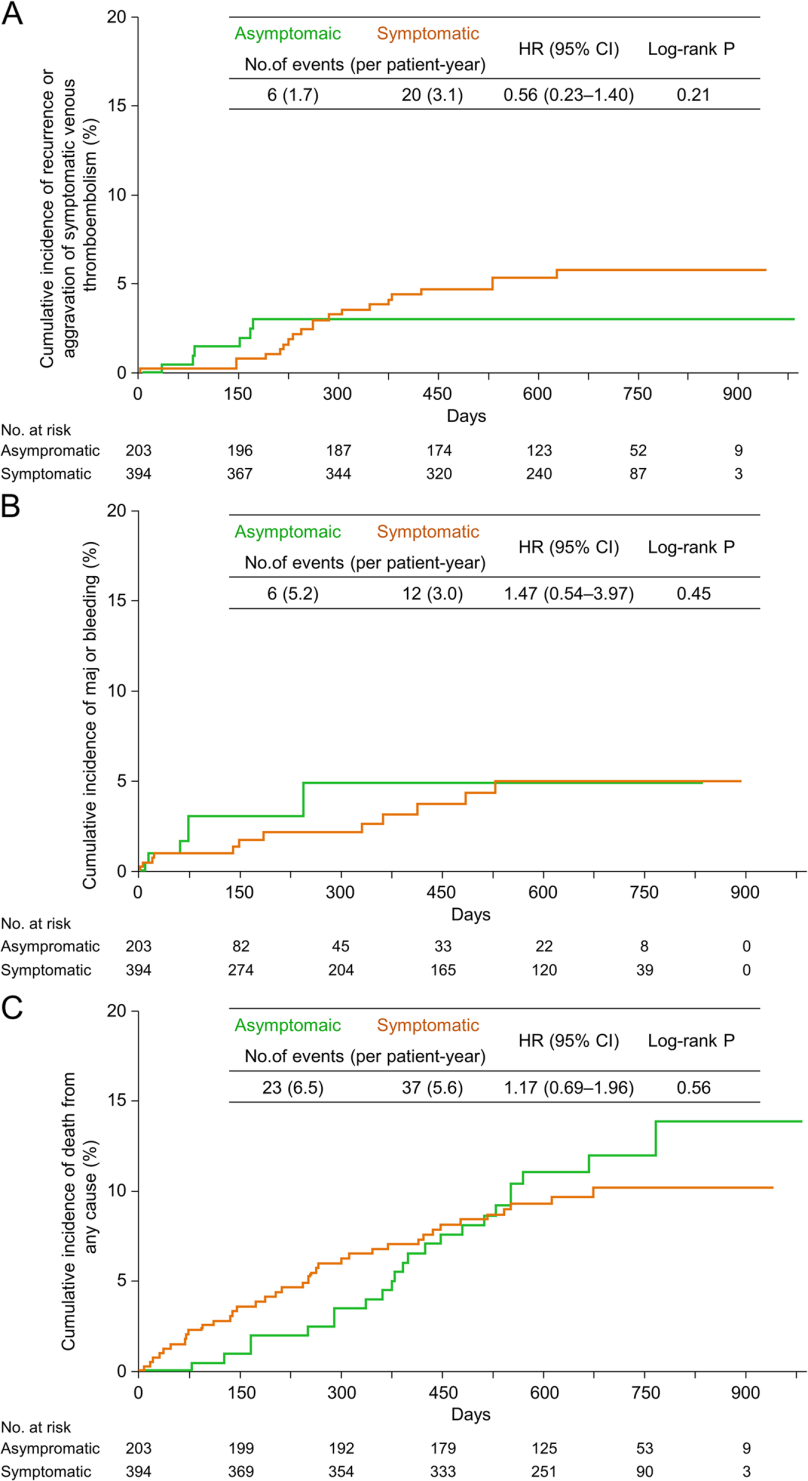
Results of clinical events in the DVT group. Kaplan–Meier curves showing the cumulative incidence of (**A**) recurrence or aggravation of symptomatic VTE, (**B**) major bleeding, and (**C**) death from any cause in the DVT group. CI, confidence interval; DVT, deep venous thrombosis; HR, hazard ratio; VTE, venous thromboembolism

No differences were observed in the recurrence or aggravation of symptomatic VTE (1.7 vs. 3.1 events per patient-year; HR, 0.56; 95% CI, 0.23–1.40; *P* = 0.21, Fig. 3A), major bleeding (5.2 vs. 3.0 events per patient-year; HR, 1.47; 95% CI, 0.54–3.97; *P* = 0.45, Fig. 3B), fatal bleeding (not observed vs. 0.5 events per patient-year; *P* = 0.21), death from any cause (6.5 vs. 5.6 events per patient-year; HR, 1.17; 95% CI, 0.69–1.96; *P* = 0.56, Fig. 3C), or clinically relevant events (9.9 vs. 9.4 events per patient-year; HR, 1.05; 95% CI, 0.69–1.61; *P* = 0.82) between the asymptomatic and symptomatic DVT groups (Table 2).

In the PE group, active cancer was a predictor of the clinically relevant events in univariate analysis (HR 4.55, 95% CI 2.72–7.62; P < 0.001). However, asymptomatic PE (HR 1.61, 95% CI 0.83–3.12; *P* = 0.16) was not a predictor. After multivariate adjustment, active cancer was an independent predictor of the clinically relevant events (HR 5.19, 95% CI 2.85–9.47; P < 0.001). However, asymptomatic PE (HR 1.44, 95% CI 0.74–2.81; *P* = 0.29) was not a predictor (Table 3). In the DVT group, active cancer (HR 3.84, 95% CI 2.53–5.82; *P* < 0.001) and lower CrCL (HR 2.46, 95% CI 1.61–3.74;0 *P* = 0.025) were predictors of clinically relevant events in univariate analysis. However, asymptomatic DVT (HR 0.95, 95% CI 0.62–1.46; *P* = 0.82) was not a predictor. After multivariate adjustment, active cancer (HR 4.12, 95% CI 2.65–6.38; *P* < 0.001) and lower CrCL (HR 2.02, 95% CI 1.20–3.42; *P* = 0.008) were independent predictors of the clinically relevant events. However, asymptomatic DVT (HR 1.13, 95% CI 0.56–1.78; *P* = 0.58) was not a predictor (Table 3).

**Table 3:** Univariate and multivariate adjustment analysis for predictors of the clinically relevant events

	PE with/without DVT				DVT			
Variables	Univariate analysis HR (95% CI)	*P*-Value	Multivariable analysis HR (95% CI)	*P*-Value	Univariate analysis HR (95% CI)	*P*-Value	Multivariable analysis HR (95% CI)	*P*-Value
Age ≥ 75 years	0.76 (0.43–1.35)	0.35			1.55 (1.03–2.32)	0.036	1.22 (0.73–2.03)	0.44
Female	0.61 (0.36–1.03)	0.06			0.81 (0.54–1.22)	0.32		
Body weight < 50 kg	1.50 (0.83–2.70)	0.18			1.92 (1.26–2.94)	0.003	0.59 (0.34–1.03)	0.06
CrCL < 50 mL/minute	0.73 (0.35–1.54)	0.41			2.46 (1.61–3.74)	0.025	2.02 (1.20–3.42)	0.008
Diabetes	1.10 (0.52–2.31)	0.81			0.65 (0.30–1.39)	0.27		
Chronic heart and lung disease	2.11 (0.96–4.65)	0.06			2.00 (0.87–4.57)	0.10		
Active cancer	4.55 (2.72–7.62)	< 0.001	5.19 (2.85–9.47)	< 0.001	3.84 (2.53–5.82)	< 0.001	4.12 (2.65–6.38)	< 0.001
Asymptomatic	1.61 (0.83–3.12)	0.16	1.44 (0.74–2.81)	0.29	0.95 (0.62–1.46)	0.82	1.13 (0.56–1.78)	0.58

Clinically relevant events were evaluated as a composite outcome, in which each component (recurrent VTE, acute coronary syndrome, ischemic stroke, death from any cause, and major bleeding events) was weighted equally. *CI *Confidence interval, *CrCL *Creatinine clearance, *DVT *Deep vein thrombosis, *HR *Hazard ratio, *PE *Pulmonary embolism

### Risk factors for clinically relevant events, recurrence or aggreviation of symptomatic VTE or major bleeding among the asymptomatic PE and DVT groups

Patients with asymptomatic PE who suffered from clinically relevant events were more likely to have chronic cardiopulmonary disease (12.0% vs. 2.3%; *P* = 0.045) and active cancer (56.0% vs. 23.3%; *P* = 0.001) than those who did not. Patients with asymptomatic DVT who suffered from clinically relevant events had a lower body weight (51.0 ± 10.5 vs. 56.3 ± 11.8 kg; *P* = 0.017) than those who did not. They were also more likely to have CrCL < 50 mL/min (39.4% vs. 20.0%; *P* = 0.025) and active cancer (54.5% vs. 21.8%; *P* < 0.001) than patients with DVT who did not suffer from clinically relevant events (Table 4). Patients with asymptomatic PE who developed clinically relevant events received rivaroxaban treatment for a shorter duration (222 [107–319] vs. 406 [173–647] days; *P* = 0.002) than patients with symptomatic PE. However, patients with asymptomatic DVT who developed clinically relevant events received rivaroxaban treatment for a longer duration than the symptomatic DVT group (170 [100–388] vs. 99 [55–223] days; *P* = 0.030) (Table 4).

**Table 4:** Patient background of clinical events in the asymptomatic PE and asymptomatic DVT groups

	Asymptomatic PE with/without DVT				Asymptomatic DVT			
	Total	Occurred clinical events	Not occurred clinical events	*P*-value	Total	Occurred clinical events	Not occurred clinical events	*P*-value
**Clinically relevant events**	*n* = 197	*n* = 25	*n* = 172		*n* = 203	*n* = 33	*n* = 170	
Age (years)	65 ± 14	67 ± 14	65 ± 15	0.47	72 ± 12	74 ± 11	71 ± 12	0.28
≥ 75 years	58 (29.4%)	7 (28.0%)	51 (29.7%)	1.00	88 (43.3%)	17 (51.5%)	71 (41.8%)	0.34
Female	94 (47.7%)	11 (44.0%)	83 (48.3%)	0.83	151 (74.4%)	24 (72.7%)	127 (74.7%)	0.49
Body weight (kg)	62.7 ± 13.8	61.3 ± 13.5	62.9 ± 13.8	0.60	55.5 ± 11.6	51.0 ± 10.5	56.3 ± 11.8	0.017
< 50 kg	33 (16.8%)	7 (28.0%)	26 (15.1%)	0.15	63 (31.0%)	15 (45.5%)	48 (28.2%)	0.06
CrCL (mL/minute)	84.5 ± 33.8	83.7 ± 37.3	84.7 ± 33.3	0.90	72.9 ± 28.4	67.8 ± 31.4	73.9 ± 27.7	0.26
< 50 mL/minute	29 (14.7%)	4 (16.0%)	25 (14.5%)	0.77	47 (23.2%)	13 (39.4%)	34 (20.0%)	0.025
Diabetes mellitus	27 (13.7%)	1 (4.0%)	26 (15.1%)	0.21	28 (13.8%)	3 (9.1%)	25 (14.7%)	0.58
Chronic heart and lung disease	7 (3.6%)	3 (12.0%)	4 (2.3%)	0.045	9 (4.4%)	3 (9.1%)	6 (3.5%)	0.16
Active cancer	54 (27.4%)	14 (56.0%)	40 (23.3%)	0.001	55 (27.1%)	18 (54.5%)	37 (21.8%)	< 0.001
D-dimer (µg/mL)	9.3 (5.3–17.5)	9.5 (6.3–16.1)	9.1 (5.1–17.5)	0.65	6.6 (2.9–11.9)	7.1 (3.4–11.1)	6.4 (2.7–12.3)	0.40
Initial rivaroxaban treatment								
Treatment duration, days								
Median (IQR)	361 (165–630)	222 (107–319)	406 (173–647)	0.002	106 (68–259)	170 (100–388)	99 (55–223)	0.030
**Recurrence or aggravation of symptomatic VTE**	*n* = 197	*n* = 6	*n* = 191		*n* = 203	*n* = 6	*n* = 197	
Age (years)	65 ± 14	60 ± 17	65 ± 14	0.35	70 ± 12	72 ± 10	70 ± 12	0.81
≥ 75 years	58 (29.4%)	1 (16.7%)	57 (29.8%)	0.67	134 (42.1%)	3 (37.5%)	131 (42.3%)	1.00
Female	94 (47.7%)	4 (66.7%)	90 (47.1%)	0.43	220 (69.2%)	6 (75.0%)	214 (69.0%)	1.00
Body weight (kg)	62.7 ± 13.7	70.0 ± 14.0	62.4 ± 13.7	0.18	57.3 ± 12.6	55.7 ± 13.5	57.3 ± 12.6	0.72
< 50 kg	33 (16.8%)	1 (16.7%)	32 (16.8%)	1.00	90 (28.3%)	4 (50.0%)	86 (27.7%)	0.23
CrCL (mL/minute)	84.5 ± 33.8	114.4 ± 44.0	83.6 ± 33.1	0.027	72.9 ± 28.4	67.5 ± 33.3	73.1 ± 28.3	0.64
< 50 mL/minute	29 (14.9%)	0 (0.0%)	29 (15.3%)	0.59	69 (22.0%)	3 (37.5%)	66 (21.6%)	0.38
Diabetes mellitus	27 (13.7%)	0 (0.0%)	27 (14.1%)	1.00	41 (12.9%)	1 (12.5%)	40 (12.9%)	1.00
Chronic heart and lung disease	7 (3.6%)	0 (0.0%)	7 (3.7%)	1.00	17 (5.4%)	1 (12.5%)	16 (5.2%)	0.36
Active cancer	51 (25.9%)	1 (16.7%)	50 (26.2%)	1.00	75 (23.6%)	2 (25.0%)	73 (23.6%)	1.00
D-dimer (µg/mL)	9.3 (5.3–17.5)	8.0 (5.1–9.3)	9.3 (5.2–17.5)	0.30	7.9 (3.5–14.2)	10.7 (3.1–16.5)	7.8 (3.5–14.2)	0.60
Initial rivaroxaban treatment								
Treatment duration, days								
Median (IQR)	361 (165–630)	216 (80–477)	362 (166–630)	0.37	106 (68–259)	170 (147–321)	173 (85–514)	0.82
**Major bleeding**	*n* = 197	*n* = 4	*n* = 193		*n* = 203	*n* = 6	*n* = 197	
Age (years)	65 ± 14	55 ± 19	65 ± 14	0.16	70 ± 12	72 ± 10	70 ± 12	0.72
≥ 75 years	58 (29.4%)	0 (0.0%)	58 (30.1%)	0.32	134 (42.1%)	3 (37.5%)	131 (42.3%)	1.00
Female	94 (47.7%)	3 (75.0%)	91 (47.2%)	0.35	220 (69.2%)	5 (62.5%)	215 (69.4%)	0.71
Body weight (kg)	62.7 ± 13.8	54.9 ± 13.5	62.8 ± 13.8	0.26	57.3 ± 12.6	54.3 ± 8.5	57.4 ± 12.7	0.50
< 50 kg	33 (16.8%)	2 (50.0%)	31 (16.1%)	0.13	90 (28.3%)	1 (12.5%)	89 (28.7%)	0.45
CrCL (mL/minute)	84.5 ± 33.8	111.9 ± 58.6	84.0 ± 33.1	0.10	72.9 ± 28.4	63.1 ± 18.7	73.2 ± 28.6	0.39
< 50 mL/minute	29 (14.9%)	0 (0.0%)	29 (15.2%)	1.00	69 (22.0%)	2 (25.0%)	67 (21.9%)	0.69
Diabetes mellitus	27 (13.7%)	0 (0.0%)	27 (14.0%)	1.00	41 (12.9%)	1 (12.5%)	40 (12.9%)	1.00
Chronic heart and lung disease	7 (3.6%)	1 (25.0%)	6 (3.1%)	0.14	17 (5.4%)	0 (0.0%)	17 (5.5%)	1.00
Active cancer	54 (27.4%)	0 (0.0%)	54 (28.0%)	0.58	75 (23.6%)	4 (50.0%)	71 (22.9%)	0.09
D-dimer (µg/mL)	9.3 (5.3–17.5)	10.3 (9.5–15.4)	9.1 (5.2–17.5)	0.53	7.9 (3.5–14.2)	7.1 (2.7–23.7)	7.9 (3.6–14.2)	0.96
Initial rivaroxaban treatment								
Treatment duration, days								
Median (IQR)	361 (165–630)	236 (41–371)	362 (168–631)	0.18	106 (68–259)	294 (38–595)	173 (86–514)	0.92

Data are shown as n (%), median (interquartile range), or mean ± standard deviation, unless otherwise stated

Clinically relevant events were evaluated as a composite outcome, in which each component (recurrent VTE, acute coronary syndrome, ischemic stroke, death from any cause, and major bleeding events) was weighted equally. *CI *Confidence interval, *CVD *Cardiovascular disease, *DVT *Deep vein thrombosis, *HR *Hazard ratio, *PE *Pulmonary embolism, *VTE *Venous thromboembolism, Data are presented as n (%) or mean ± SD. *CrCL *Creatinine clearance, *DVT *Deep vein thrombosis, *IQR *Interquartile range, *PE *Pulmonary embolism, *SD *Standard deviation

In the asymptomatic PE group, patients with recurrence or aggravation of symptomatic VTE had a significantly higher CrCL (114.4 ± 44.0 vs. 83.6 ± 33.1 mL/minute; *P* = 0.027) than those without it. However, no other differences were observed. There were also no significant differences between the two groups of patients who did and didn't experience major bleeding. In the asymptomatic DVT group, there were no significant differences in between the patients with and whout recurrence or aggravation of symptomatic VTE, or major bleeding (Table 4).

### Relationship between active cancer and clinical events in the asymptomatic PE and DVT groups

In the asymptomatic PE group, active cancer had no effect on VTE recurrence (1.9% vs. 3.5%; *P* = 1.00) (Table 5). While the occurrence of major bleeding was not significantly different (0.0% vs. 2.8%; *P* = 0.58) between the two groups, the occurrences of all-cause death (25.9% vs. 2.1%; *P* < 0.001) and clinically relevant events (25.9% vs. 7.7%; *P* = 0.001) were significantly higher in the group with active cancer (Table 5). In the asymptomatic DVT group, active cancer had no effect on VTE recurrence (1.8% vs. 3.4%; *P* = 1.00). However, major bleeding (7.3% vs. 1.4%; *P* = 0.047), all-cause death (29.1% vs. 4.7%; *P* < 0.001), and clinically relevant events (32.7% vs. 10.1%; *P* < 0.001) were all significantly higher in the group with active cancer (Table 5).

**Table 5:** Clinical outcomes in patients with and without active cancer within the asymptomatic VTE patients

	Asymptomatic PE with/without DVT				Asymptomatic DVT			
	Total	With active cancer	Without active cancer	*P*-value	Total	With active cancer	Without active cancer	*P*-value
	*n* = 197	*n* = 54	*n* = 143		*n* = 203	*n* = 55	*n *= 148	
Recurrence or aggravation of symptomatic VTE	6 (3.0%)	1 (1.9%)	5 (3.5%)	1.00	6 (3.0%)	1 (1.8%)	5 (3.4%)	1.00
Recurrence or aggravation of symptomatic PE	3 (1.5%)	1 (1.9%)	2 (1.4%)	1.00	1 (0.5%)	0 (0.0%)	1 (0.7%)	1.00
Recurrence or aggravation of symptomatic DVT	4 (2.0%)	0 (0.0%)	4 (2.8%)	0.58	5 (2.5%)	1 (1.8%)	4 (2.7%)	1.00
Acute coronary syndrome	0 (0.0%)	0 (0.0%)	0 (0.0%)	-	1 (0.5%)	0 (0.0%)	1 (0.7%)	1.00
Ischemic stroke	4 (2.0%)	2 (3.7%)	2 (1.4%)	0.30	0 (0.0%)	0 (0.0%)	0 (0.0%)	-
Death from any cause	17 (8.6%)	14 (25.9%)	3 (2.1%)	< 0.001	23 (11.3%)	16 (29.1%)	7 (4.7%)	< 0.001
Death related to VTE	2 (1.0%)	1 (1.9%)	1 (0.7%)	0.47	1 (0.5%)	0 (0.0%)	1 (0.7%)	1.00
Death related to CVD	3 (1.5%)	1 (1.9%)	2 (1.4%)	1.00	1 (0.5%)	0 (0.0%)	1 (0.7%)	1.00
Major bleeding	4 (2.0%)	0 (0.0%)	4 (2.8%)	0.58	6 (3.0%)	4 (7.3%)	2 (1.4%)	0.047
Minor bleeding	7 (3.6%)	3 (5.6%)	4 (2.8%)	0.40	14 (6.9%)	3 (5.5%)	11 (7.4%)	0.76
Fatal bleeding	0 (0.0%)	0 (0.0%)	0 (0.0%)	-	0 (0.0%)	0 (0.0%)	0 (0.0%)	-
Clinically relevant events	25 (12.7%)	14 (25.9%)	11 (7.7%)	0.001	33 (16.3%)	18 (32.7%)	15 (10.1%)	< 0.001

Data are shown as n (%), unless otherwise stated

Clinically relevant events were evaluated as a composite outcome, in which each component (recurrent VTE, acute coronary syndrome, ischemic stroke, death from any cause, and major bleeding events) was weighted equally. *CVD *Cardiovascular disease, *DVT *Deep vein thrombosis, *PE *Pulmonary embolism, *VTE *Venous thromboembolism

## Discussion

This study had three major findings: First, while there were no differences in age, sex, body weight, or renal function, patients with asymptomatic PE had better vital signs and lower severity with a lower level of RV dysfunction but a higher prevalence of proximal DVT than patients with symptomatic PE. Patients with asymptomatic DVT were older and often female with lower body weight and renal function. However, they had a higher prevalence of distal DVT relative to those with symptomatic DVT. In both groups, active cancer and history of recent surgery were more common in asymptomatic patients than in symptomatic patients. Second, regarding rivaroxaban treatment, asymptomatic PE patients mostly had an intensive rivaroxaban treatment with a modestly longer treatment duration than symptomatic PE patients. In contrast, asymptomatic DVT patients more often received an off-label reduced dose of rivaroxaban treatment with a significantly shorter treatment duration than symptomatic DVT patients. Third, in the PE and DVT groups, the incidences of recurrence or aggravation of symptomatic VTE, major bleeding, or clinically relevant events did not differ between the asymptomatic and symptomatic groups.

### Characteristics and clinical outcomes of patients with asymptomatic PE

We have clarified the differences in patient characteristics between patients with asymptomatic and symptomatic PE who received rivaroxaban treatment in Japan. No differences were observed in age, sex, body weight, or renal function between the asymptomatic and symptomatic PE groups. However, the asymptomatic PE patients were at a lower risk of PE, which was reflected by better vital signs, less frequent massive PEs, and lower RV dysfunction as assessed by the RV/LV diameter ratio. Unexpected and incidentally detected PE is reported in 3.3–5.0% of cancer patients and 1–2% of all thoracic CT scans [7, 18, 19]. Our data indicated that the rate of active cancer in the asymptomatic group was 27%, which was higher than that in the symptomatic PE group. 11–27% of all incidentally discovered PEs are confined to subsegmental vessels [9, 10, 20]. In this study, approximately 80% of the asymptomatic PE patients had non-massive PE, suggesting that some patients had subsegmental PE. The effects of treating incidental PE by direct oral anticoagulants (DOAC) have not yet been evaluated in large prospective studies. There are some real-world data supporting the treatment of incidentally detected PE in patients with known VTE risk factors such as cancer because these patients have been reported to have high rates of recurrent VTE and mortality [10], especially those who had not received anticoagulation therapy [11]. Unfortunately, Japanese guidelines have not stated a treatment strategy for particular patients with asymptomatic PE and concomitant cancer or those without cancer thus far [2]. Nonetheless, the 2019 European Society of Cardiology guidelines recommend that patients with incidentally detected PE and concomitant cancer should be managed similarly to those with symptomatic PE, also stating that no firm data exist [21], as similarly recommended by the American Society of Clinical Oncology (informal consensus, moderate strength recommendations) [22]. The higher proportion of proximal DVT and active cancer in the asymptomatic PE group in this study could be a consequence of the physicians’ motivation to prescribe rivaroxaban treatment to improve the prognosis even in these asymptomatic PE patients. Asymptomatic PE can also lead to pulmonary hypertension. In recent years, the prevalence of CTEPH after symptomatic acute PE treatment has been reported to be 3.8–5.4% [23, 24]. Theoretically, asymptomatic PE patients might be underdiagnosed and thus not receive anticoagulant therapy, leading to an increased risk of developing CTEPH. The prognoses of incidentally discovered PE and symptomatic PE are similar [25].

Hence, the American College of Chest Physicians (Grade 2B) recommends treating patients with incidentally discovered PE and symptomatic PE similarly to those with symptomatic VTE [26]. Therefore, in the real-world setting, physicians could have decided to prescribe the initial on-label dose of rivaroxaban (30 mg) to most patients with asymptomatic PE. However, the dose might have been reduced in some patients with low-VTE risk, such as subsegmental PE. Our study also demonstrated equivalent incidences of recurrent VTE, major bleeding, and a composite outcome between asymptomatic and symptomatic PE groups receiving rivaroxaban treatment. Major bleeding events were numerically, and minor bleeding events were significantly lower in the asymptomatic than the symptomatic PE group. None of the asymptomatic PE patients experienced fatal bleeding. This supports the evaluation that the benefit of improved prognosis outweighs the risk of bleeding burden from rivaroxaban treatment, even in most patients with asymptomatic PE.

### Characteristics and clinical outcomes of patients with asymptomatic DVT

In our study, distal DVT was observed in 73.9% of patients with asymptomatic DVT. This was higher than that observed in patients with symptomatic DVT. Patients with asymptomatic DVT had a higher incidence of recent surgery and trauma. These results suggest that DVT was incidentally detected after general or orthopedic surgery in most patients with asymptomatic DVT. The detection of asymptomatic distal DVT by postoperative screening has increased in recent years [12]. Asymptomatic distal DVT is considered less risky than symptomatic peripheral DVT for VTE recurrence [13]. In a prospective blinded study, the incidence of PE in untreated distal DVT patients was as low as 1.6% [27]. Distal DVT is associated with fewer PE recurrences than proximal DVT [28]. A double-blind, randomized controlled study evaluating low-molecular-weight heparin treatment of symptomatic distal DVT demonstrated no benefit from anticoagulation in preventing worsening DVT or symptomatic PE (3% vs. 5%; *P* = 0.54) and only increased hemorrhagic complications (4% vs. 0%; *P* = 0.0255) [29]. Therefore, there is no evidence supporting the benefit of anticoagulant therapy, such as heparin or warfarin, in patients with asymptomatic DVT or distal DVT. The present study highlights the potential of DOAC treatment in asymptomatic DVT patients as the annual incidence of recurrent VTE in the asymptomatic DVT group did not differ from that in the symptomatic DVT group under rivaroxaban treatment (1.7 vs. 3.1 events per 100 patient-year; *P* = 0.21).

The annual recurrent VTE incidence reported in this study is similar to that reported for symptomatic Japanese and global VTE patients who had been treated with rivaroxaban in the J-EINSTEIN-PE/DVT [30] (1.8%) and EINSTEIN-PE/DVT [31, 32] (1.8%) studies, respectively.

Additionally, the incidence of major bleeding in the asymptomatic DVT group was not different from that in the symptomatic DVT group (5.2 vs. 3.0 events per 100 patient-year; *P* = 0.45). However, this incidence was modestly higher as compared with the annual rates reported in the J-EINSTEIN-PE/DVT (0.0%) and EINSTEIN-PE/DVT (1.1%) studies. Therefore, the risks and benefits should be carefully considered when initiating rivaroxaban in asymptomatic DVT patients, especially in patients with a high risk of bleeding from cancer. Major bleeding events in this study were mostly due to concomitant active cancer (67%, 4/6 patients suffering from major bleeding, Table 4). A retrospective observational study of Japanese VTE patients treated with DOACs [33] also reported that bleeding events due to active cancer complications should be considered more important than VTE-related events in patients with active cancer complicated with VTE. Therefore, in the real-world setting of this study, it is speculated that the asymptomatic DVT group as compared with the symptomatic group was more often administered off-label underdosed rivaroxaban by physicians, with a shorter treatment duration. This was possibly due to the high bleeding and low VTE risk in this group, which was also characterized by older age, a higher female sex ratio, lower body weight and CrCL, as well as a higher proportion of distal DVT and active cancer.

### Clinical implications

Our data may provide a basis for using rivaroxaban to treat and prevent the recurrence of VTE in patients with asymptomatic PE (with or without DVT) and DVT-only. Patients with asymptomatic PE displayed background characteristics similar to those with incidentally detected PE as reported previously; i.e., the PE severity involved mostly low-risk non-massive and occasionally sub-massive PEs, but the concomitant cancer rate was high. Despite the use of intensive rivaroxaban treatment in the asymptomatic PE group, clinically relevant events, including VTE recurrence and mortality in this group were similar to those in the symptomatic PE group. In addition, major bleeding and fatal bleeding events were low. Our data suggest that intensive rivaroxaban treatment can be used for the prophylaxis of VTE and VTE-related complications, even in patients with asymptomatic PE, with or without concomitant cancer, as recommended previously for patients with incidentally detected PE [21, 22, 26].

The asymptomatic DVT group also demonstrated a higher rate of cancer incidence, similar to the PE group. Concomitant active cancer was one of the risk factors for clinically relevant events, mostly driven by cancer-related death rather than bleeding or VTE-related death in both the PE and DVT groups. Our study strongly recommends that concomitant active cancer should be carefully managed in both the PE and DVT-only groups. Notably, the asymptomatic DVT group had a higher risk of bleeding (related to older age, female sex, lower body weight, and lower renal function) and higher incidence of distal DVT than the asymptomatic PE group. Due to this, asymptomatic DVT patients were more likely to receive an off-label underdose rivaroxaban with a shorter treatment duration than that received by the symptomatic DVT and the symptomatic/asymptomatic PE group. The incidence of recurrent VTE was lowest in this group (1.7 events per patient-year). However, major bleeding events were highest in the asymptomatic DVT group among the four groups (5.2 vs. 3.0 events per patient-year for symptomatic DVT, 1.9 for asymptomatic PE, and 2.7 for symptomatic PE, respectively). Therefore, the decision to initiate rivaroxaban should be carefully made while considering the risks and benefits for patients with asymptomatic DVT. Once physicians decide to initiate rivaroxaban, an underdosed rivaroxaban with treatment duration adjustment based on careful monitoring of the bleeding and VTE risks could be an option for such patients [34].

### Limitations

This study has some limitations. First, the sample size was relatively small, and the number of clinical events was also limited because this study only included Japanese patients. The reported incidence rates of PE and DVT in Western countries [3] seem to be higher as compared to those reported for the Japanese population [35]. This difference could be attributed to variations in the underlying causes. Factor V Leiden mutation is a common cause of DVT among Western individuals [36], while protein S deficiency is more frequently associated with DVT in Japanese patients [37], with Factor V Leiden being rarely reported in Japan. It is also important to note that the genetic predisposition to venous thrombosis differs between Japanese and Western populations. Therefore, it remains uncertain whether our study findings can be directly applied to individuals in Western countries. Second, selection bias could have occurred. Patients enrolled in this study received rivaroxaban at the discretion of their physicians; patients who were diagnosed with VTE but did not receive anticoagulation were not included in this study. However, their absence must be considered. Thus, selection bias in enrolling patients must be carefully considered when interpreting the results of this study. Finally, information was lacking regarding the enrolled patients. In particular, the lack of information on the detailed location of thrombi in pulmonary emboli and the clinical stage of patients with malignant tumors may have led to inadequate analyses.

## Conclusions

This study has clarified the characteristics of patients with asymptomatic PE with and without DVT and those with DVT only. The asymptomatic PE and DVT groups were more likely to have active cancer. However, they experienced lower severity of PE and DVT as compared with patients with symptomatic PE and DVT-only there were more cases of non-massive PE in the asymptomatic PE group and more cases of distal DVT in the asymptomatic DVT group. Patients with asymptomatic DVT had the highest risk of bleeding among the four groups. They were also most often characterized by old age, female sex, low body weight, and low renal function. The real-world composite adverse event rate of rivaroxaban treatment was similar for patients with asymptomatic and symptomatic PE or DVT, as demonstrated using physician-adjusted dose and duration. This suggests a potential for safe dose- and duration-adjusted rivaroxaban treatment for asymptomatic VTE patients.

## Data Availability

The deidentified participant data will not be shared.

## Abbreviations

ACS: Acute coronary syndrome
CI: Confidence interval
CrCl: Creatinine clearance
CT: Computed tomography
CTEPH: Chronic thromboembolic pulmonary hypertension
CVD: Cardiovascular disease
DOAC: Direct oral anticoagulants
DVT: Deep vein thrombosis
HR: Hazard ratio
IQR: Interquartile range
IRB: Institutional Review Board
LV: Left ventricle
mITT: Modified intention-to-treat
NSAIDs: Nonsteroidal anti-inflammatory drugs
PCPS: Percutaneous cardiopulmonary support
PE: Pulmonary embolism
RV: Right ventricle
SD: Standard deviation
SpO_2_: Oxygen saturation
VTE: Venous thromboembolism
